# Physicians’ decreased tendency to choose palliative care for patients with advanced dementia between 1999 and 2015

**DOI:** 10.1186/s12904-021-00811-5

**Published:** 2021-07-26

**Authors:** Saila Haapasalmi, Reetta P. Piili, Riina Metsänoja, Pirkko-Liisa I. Kellokumpu-Lehtinen, Juho T. Lehto

**Affiliations:** 1 Faculty of Medicine and Health Technology, Tampere University, Tampere, Finland; 2 Palliative Care Centre and Department of Geriatrics, Tampere University Hospital, Tampere, Finland; 3 Tays Hatanpää Hospital, Hatanpäänkatu 24, T-Building, 4th floor, 33900 Tampere, Finland; 4 Palliative Care Centre and Tays Cancer Centre, Department of Oncology, Tampere University Hospital, Tampere, Finland; 5 Faculty of Social Sciences, Tampere University, Tampere, Finland; 6 Tays Cancer Centre, Department of Oncology, Tampere University Hospital, Tampere, Finland

**Keywords:** Decision-making, End-of-life care, Dementia, Physician

## Abstract

**Background:**

Physicians’ decision-making for seriously ill patients with advanced dementia is of high importance, especially as the prevalence of dementia is rising rapidly, and includes many challenging ethical, medical and juridical aspects. We assessed the change in this decision-making over 16 years (from 1999 to 2015) and several background factors influencing physicians’ decision.

**Methods:**

A postal survey including a hypothetical patient-scenario representing a patient with an advanced dementia and a life-threatening gastrointestinal bleeding was sent to 1182 and 1258 Finnish physicians in 1999 and 2015, respectively. The target groups were general practitioners (GPs), surgeons, internists and oncologists. The respondents were asked to choose between several life-prolonging and palliative care approaches. The influence of physicians’ background factors and attitudes on their decision were assessed.

**Results:**

The response rate was 56%. A palliative care approach was chosen by 57 and 50% of the physicians in 1999 and 2015, respectively (*p* = 0.01). This change was statistically significant among GPs (50 vs 40%, *p* = 0.018) and oncologists (77 vs 56%, *p* = 0.011). GPs chose a palliative care approach less often than other responders in both years (50 vs. 63% in 1999 and 40 vs. 56% in 2015, *p* < 0.001). In logistic regression analysis, responding in 2015 and being a GP remained explanatory factors for a lower tendency to choose palliative care. The impact of family’s benefit on the decision-making decreased, whereas the influence of the patient’s benefit and ethical values as well as the patient’s or physician’s legal protection increased from 1999 to 2015.

**Conclusions:**

Physicians chose a palliative care approach for a patient with advanced dementia and life-threatening bleeding less often in 2015 than in 1999. Specialty, attitudes and other background factors influenced significantly physician decision-making. Education on the identification and palliative care of the patients with late-stage dementia are needed to make these decisions more consistent.

## Introduction

Affecting approximately 50 million people worldwide, the expanding epidemic of dementia has become one of the greatest social and medical challenges [1]. Dementia is usually a consequence of a progressive disease, such as Alzheimer’s disease, without curative therapies available. Therefore, it is currently one of the most common causes of disability, dependency and death among older people [2].

Many ethical, legal, religious, medical and psychological aspects influence end-of-life (EOL) decision-making; thus, decisions seem to vary, depending on the treating physician [3–6]. For example, age and specialty of a physician has been shown to influence physicians’ decision-making [7]. As there is growing evidence of the benefits of palliative care both in patient’s physical and psychological welfare and increase in education of palliative medicine worldwide after the early 2000, it can be assumed that physicians are more willing to choose palliative care approach to their patients today [8, 9].

Better understanding of the prognosis and expected complications of advanced dementia is likely to reduce potentially burdensome interventions of questionable benefit near the EOL [10, 11]. Even though identical decisions by all physicians in complex clinical situations may not be realistic, the basic principles in decision-making should be consistent and the significant palliative care needs of people with advanced dementia should be met equally. Due to population’s aging, physicians with different specialities are taking care of more patients with age-related conditions such as dementia. For example, cancer is found more progressively at older age making it essential for oncologists to have knowledge about the vulnerability of the patients with dementia and to integrate geriatric assessment to their patients’ treatment planning [12–15]. In addition, advanced stages and treatment complications of comorbidities among patients with dementia force many specialists to make decisions concerning palliative or life-prolonging therapies in complex situations, where the benefits and harms have to be considered in the context of patient’s advanced dementia.

The late stage of dementia is characterized by a loss of most cognitive functions, incontinency and impairment in daily activities such as eating and walking [2, 16, 17]. Emergency department visits and hospital admissions are frequent among patients with advanced dementia [2, 10, 18–20]. These patients are vulnerable to the adverse effects of the transition from a nursing home to a hospital, and hospitalizations may lead to burdensome treatments of uncertain benefit and undertreatment of pain and other symptoms [19, 21, 22]. According to the Recommendation on the Provision and Improvement of Palliative Care Services in Finland, 43% of older people living in nursing homes used acute services within three months before their death in 2016 [22].

In spite of the progressive nature of dementia, it has only recently been recognized as a life-limiting condition that benefits from palliative care [21, 23, 24]. EOL care for patients with dementia has been gaining increasing interest of researchers and health care professionals and many barriers to palliative care for advanced dementia have been recognized [16, 25]. Palliative care interventions in advanced dementia have been carried out especially in Europe and United States and there is some evidence, that hospice and palliative care enrolment of people with dementia has slightly increased [26–29]. It seems, though, that EOL of care for people with dementia varies considerably, and for instance in Finland research on this field is still limited [30, 31].

Advance care planning (ACP) with discussions of goals of care for the acute complications and a plan for EOL care has been identified as an effective strategy to improve EOL care of people with dementia [3, 31–33]. It should take place early while patients have sufficient mental capacity to consider their preferences and make decisions about their future. However, only approximately 40% of people with dementia undertake ACP [33–37]. Therefore, physicians are often forced to make critical decisions for patients with advanced dementia in acute situations [4]. The current coronavirus (COVID-19) pandemic, which has especially affected the older, frail population, particularly in nursing homes has also revealed the importance of valuable decision-making and ACP for these patients [38–40].

The aim of this study was to examine whether physicians’ decision-making concerning a patient with advanced dementia changed over 16 years (from 1999 to 2015) and to identify possible factors explaining the differences relating to these decisions. The findings are relevant in planning post- and undergraduate education concerning dementia and EOL care of aging population.

## Methods

Two cohorts of Finnish physicians answering a postal survey with a questionnaire and a cover letter formed the study sample. The first cohort answered a questionnaire in 1999, whereas the second completed similar one in 2015. Both cohorts included 500 general practitioners (GPs), 300 surgeons and 300 internists who were randomly selected from the register of the Finnish Medical Association as well as Finnish oncologists (*n* = 82 in 1999 and *n* = 158 in 2015). Two reminders were sent to non respondents. The cover letter included an introduction to the study and an assurance of anonymity and voluntariness. This study was approved by the Regional Ethics Committee of Tampere University Hospital, Finland (R15101).

The questionnaire included altogether seven hypothetical patient scenarios (six patients with cancer and one with dementia). The patient scenarios were exactly the same with same wordings in both years. In addition to the patient scenarios, several questions regarding factors influencing physicians’ decisions as well as their background, life values and attitudes were presented. Respondents were instructed to answer the questions in the given order and not to change their answers once given. Attitudes towards multiple ethical and moral aspects were assessed with a 100 mm visual analogue scale, with responses ranging from “definitely agree” (0 mm) to “definitely disagree” (100 mm). Changes in these attitudes between 1999 and 2015 concerning a cancer patient have been reported earlier [6]. A pilot study was done in January 1999 after which the questionnaire has been validated with Finnish physicians and used in studies concerning cancer patient scenarios and responses to dementia patient scenario in 1999 [3, 6, 41, 42]. Responses to dementia patient scenario in 2015 haven’t been published earlier.

Here we report the patient scenario of a patient with advanced dementia. The patient scenario used was exactly the same in 1999 and 2015. The patient scenario involved an 82-year-old man suffering from progressive dementia. He had been diagnosed as suffering from Alzheimer’s disease for three years. He was brought to the emergency department at 2 am with life-threatening gastrointestinal bleeding. He lived in a nursing home, had urinary and faecal incontinence, needed help washing and dressing and could not identify his daughter. His blood pressure was 70/40 mm Hg, and his heart rate 120 beats/min. The patient could not communicate, and neither his family nor his physician could be reached. The nurse’s aide who accompanied him to the emergency department was not familiar with the patient. There was no information available about his wishes or those of his family concerning treatment in this kind of situation. The respondents were asked to choose one of the given treatment decisions: a) palliative care b) active care or c) intensive care. The treatment options were explained as follows: (a) palliative care: good nursing, sufficient medications for pain and other symptoms, and intravenous hydration only when considered to relieve the patient’s symptoms; (b) active care: use of antibiotics, intravenous hydration or blood transfusions aimed at saving the patient’s life in a life-threatening condition and (c) intensive care: moving the patient to an intensive care unit. After respondents were asked about the treatment decision, a Likert-type scale was presented to evaluate the influence of different factors (patient’s benefit, family’s benefit, patient’s legal protection, physician’s legal protection, ethical values, patient’s age, cost of care and patient’s social status) on their decision (from 1—very little influence to 5—very much influence) [3].

### Statistical analysis

The answers to the hypothetical patient scenario were re-categorized dichotomously. The conversion was conducted as follows (responses shown in brackets): “choosing palliative care” (a) and “not choosing palliative care” (b and c). The answers on the 5-point Likert scale concerning the influence of different factors were converted to the following 2-point scale: 1–3 for “not much influence” and 4–5 for “much influence”. Dichotomous variables were tested using the Pearson chi-squared test. Continuous variables were tested using an independent samples t-test or the Mann–Whitney U-test, if the data were not normally distributed. Two-sided p-values of less than 0.05 were considered as statistically significant.

### Logistic regression analysis

The model explaining the decision to choose active/intensive care was created using forward stepwise logistic regression. Background factors, life values and attitudes were all included in the model, except the work place because it had a significant association with physician groups (*p* < 0.001), e.g. surgeons working in hospitals and GPs in outpatient units. When responses to “People should pay costs of factitious diseases by themselves” entered the model, the p-value for the year of the survey changed from 0.050 to 0.145 outside of the model and it didn’t enter. Responses to “People should pay cost of factitious diseases by themselves” were chosen to be left out because it did not independently explain the physicians’ decision to choose palliative care (*p* = 0.133). On the contrary, the year of study was independently associated with the decision to choose palliative care (*p* = 0.025). The p-value for significance was set at 0.10 to enter and 0.15 to remove from the model.

The data analysis was performed using IBM SPSS Statistics for Windows, V.25.0.

## Results

Altogether, 1373 valid responses were received, giving a response rate of 56%. Response rates according to year of response were 62% in 1999 and 51% in 2015. Table 1 shows the characteristics of the respondents according to physician group and year of response. In 2015, respondents more often were women (*p* < 0.001), were older (*p* < 0.001) and had longer working experience (*p* < 0.001). The response rate increased among oncologists and decreased among all the other physician groups between the study years. The highest response rate was achieved among GPs (63%) in 1999 and oncologists (66%) in 2015. About half of the respondents worked at hospitals in both years (47% in 1999 and 57% in 2015) and most of them were married (77% in 1999 and 80% in 2015).

**Table 1:** Characteristics of the participants

	*Surgeons*				*Internists*				*GPs*				*Oncologists*					***Total***			
	*1999*		*2015*		*1999*		*2015*		*1999*		*2015*		*1999*		*2015*			*1999*		*2015*	
Number (% of total)	175	(24)	142	(22)	184	(25)	153	(24)	316	(43)	245	(38)	54	(7)		104	(16)	**729**	**(100)**	**644**	**(100)**
Response rate, %	58		47		61		47		63		49		51			66		**62**		**51**	
Female, n (%)	33	(19)	47	(33)	60	(33)	81	(53)	170	(55)	173	(71)	30	(56)		85	(82)	**293**	**(41)**	**386**	**(60)**
Mean age (range)	48	(33–66)	51	(33–64)	48	(32–70)	52	(33–65)	42	(25–63)	47	(25–65)	46	(35–61)		48	(32–67)	**45**	**(25–70)**	**50**	**(25–67)**
Work place^a^																					
Outpatient unit	1	(1)	2	(1)	15	(9)	15	(10)	242	(78)	208	(86)	2	(4)		4	(4)	**260**	**(37)**	**229**	**(36)**
Hospital	146	(85)	124	(88)	123	(71)	122	(82)	33	(11)	24	(10)	44	(83)		91	(88)	**346**	**(49)**	**361**	**(57)**
Other	24	(14)	15	(11)	35	(20)	12	(8)	35	(11)	10	(4)	7	(13)		8	(8)	**101**	**(14)**	**45**	**(7)**
Years from graduation, median (range)^b^	22	(2–42)	26	(7–42)	21	(7–41)	26	(8–42)	16	(1–35)	21	(0–40)	18	(9–34)		22	(7–40)	**19**	**(1–42)**	**23**	**(0–42)**
Married, n (%)	140	(81)	119	(84)	142	(79)	124	(81)	228	(73)	198	(81)	45	(83)		71	(71)	**555**	**(77)**	**512**	**(80)**

*GP* general practitioner

^a^ For 32 participants working place was not available

^b^ For 19 participants year of graduation was not available

Respondents chose a palliative care approach in the patient scenario less often in 2015 than in 1999. This change was statistically significant among the GPs and oncologists (Table 2). GPs chose palliative care less frequently than other physician groups (*p* < 0.001) in both years. Specifically, 50% (*n* = 155) and 40% (*n* = 92) of GPs chose palliative care in 1999 and 2015, respectively, whereas 63% (*n* = 255) and 56% (*n* = 211) of other physicians chose palliative care in 1999 and 2015, respectively. In both years, a palliative care approach was chosen more often by physicians working in hospitals (58%) than those working in outpatient units (48%) (*p* = 0.005). Only seven (1%) respondents chose intensive care in 1999 and four (0,6%) in 2015.

**Table 2:** Number and proportion (%) of responders choosing palliative care approach for the dementia patient

*Choosing palliative care*	1999		2015		*P*-value*
Surgeons	108	(62%)	78	(57%)	0.366
GPs	155	(50%)	92	(40%)	0.018
Internists	107	(59%)	80	(56%)	0.566
Oncologists	40	(77%)	53	(56%)	0.011
**Total**	**410**	**(57%)**	**303**	**(50%)**	**0.010**

*GP* General Practitioner

^*^ Pearson Chi-square

The results of the logistic regression analysis are presented in Table 3. Answering the questionnaire in 2015 remained a significant independent factor explaining the physicians’ decision to choose active/intensive care for the dementia patient in the regression analysis. In addition, being a GP or an internist was associated with an increased likelihood of choosing active/intensive care compared to oncologists. Older age was associated with decreased likelihood of choosing active/intensive care.

**Table 3:** Different background factors and attitudes explaining the decision to choose active or intensive care (*n* = 495) over palliative care (*n* = 574) in forward logistic regression analysis

	n	OR	(95% CI)	*p*
Year of the survey				0.033
1999	575	ref		
2015	494	1.37	(1.03, 1.84)	
Physician groups				0.017
Oncologists	114	ref		
Surgeons	250	1.41	(0.87, 2.28)	0.162
Internists	262	1.65	(1.02, 2.66)	0.042
GPs	443	1.98	(1.26, 3.10)	0.003
Age	1069	0.96	(0.94, 0.97)	< 0.001
Taking care of a family member in end-of-life				0.077
No	411	ref		
Yes	658	0.78	(0.60, 1.03)	
Importance of family				0.060
Important	1042	ref		
Not important	27	2.16	(0.97, 4.84)	
Withdrawal of life-sustaining treatments is reprehensible (VAS^a^)	1069	0.91	(0.86, 0.96)	0.001
Advance directives have been helpful in my decisions (VAS)	1069	0.94	(0.89, 0.99)	0.016
It is waste of resources to treat patients over 80 years of age in intensive care units (VAS^a^)	1069	1.07	(1.02, 1.13)	0.007

*GP* General Practitioner, *ref.* reference

^a^ VAS, visual analogue scale (0 definitely agree, 10 definitely disagree). One unit is equivalent to 10 mm on a 100-mm VAS

Table 4 summarizes physicians’ answers regarding different factors influencing their decision. Family’s benefit was less influential on the physicians’ decision-making in 2015 than in 1999, whereas the influence of patient’s benefit and ethical values as well as the patient’s or physician’s legal protection significantly increased from 1999 to 2015. In both years, about one third of the respondents considered patient’s age to have much influence on their decision, while social status or cost of care did not have much influence on the decision.

**Table 4:** Proportion of the physicians who stated different factors to have much influence on their decisions concerning the care of the dementia patient

Having much influence	1999		2015		*P*-value*
Patient’s benefit	647	(89%)	590	(93%)	0.026
Family’s benefit	247	(34%)	134	(21%)	< 0.001
Patient’s legal protection	499	(69%)	505	(80%)	< 0.001
Physician’s legal protection	350	(48%)	397	(62%)	< 0.001
Ethical values	577	(80%)	565	(89%)	< 0.001
Patient’s age	271	(37%)	221	(35%)	0.344
Costs of care	66	(9%)	50	(8%)	0.423

^*^Pearson Chi-square

## Discussion

In our study, Finnish physicians were more unlikely to choose a palliative care approach in 2015 than in 1999 for a patient with an advanced dementia and a life-threatening gastrointestinal bleeding*.* The overall tendency to choose a more active approach for a dementia patient in 2015 is opposite to the findings in our previous study of changes in physicians’ decision-making concerning a terminally ill cancer patient [6].

We have to honestly acknowledge that our study has several limitations. There is a possibility that the decisions would have been different in a real-life situation. Nevertheless, we assume that the answers do reflect real-life decision-making and reveal true changes in physicians’ attitudes. Our response rate (56%) needs to be acknowledged, because of possible nonresponse bias. On the other hand, our study population was a large and representative sample of Finnish physicians, and the long follow-up period allows for the detection of changes in decision-making. Both samples were randomly selected from the register of the Finnish Medical Association. Due to the privacy protection laws in Finland, we were not allowed to store the personal data of the respondents from the year 1999. Therefore, it is possible that some of the respondents have answered to the survey in both years. However, it is unlikely that this formed a major proportion of our respondents due to the long study period and a large number of physicians working in Finland (altogether 16 020 in 2000 and 20 970 in 2016) [43]. There are differences between the respondent groups, as shown in Table 1, but these differences reflect the changes that have happened after 1999 among Finnish physicians. Between the years 2000 and 2015, the proportion of female physicians has increased from little less than 50% to 60% and the number of older physicians (aged 55 to 60 years) has increased from slightly over 2 000 to more than 5 000 physicians in Finland [43]. Finally, only one case scenario in our whole questionnaire represented a patient with dementia, while all the others were cancer patients. This reflects the overall understanding of palliative care as a treatment modality concerning mainly cancer patients in 1999. Although this might have influenced to the responses, we wanted to maintain the questionnaire exactly the same in 2015 to allow comparison between the study years.

We have to declare that the choices between palliative or life-prolonging therapy in our study were multidimensional, and no strictly right or wrong answers were presented or preselected. However, the patient in our scenario had common features of late-stage dementia and could be regarded as severely frail utilizing the Clinical Frailty Scale (CFS), which has been validated as an adverse outcome predictor in hospitalized older patients [44, 45]. Therefore, we suggest that the patient would have benefited most from good symptomatic care with a palliative intent instead of more aggressive therapeutic options. The benefits of integrated palliative care concomitantly with active treatment are shown in cancer and may be reasonable also in dementia with rather uncertain prognosis [46]. This could have led some of our respondents to choose time-limited active approach, but with an intent to combine careful symptom-centred care at the same time.

Choosing a palliative care approach less often in 2015 than in 1999, was somewhat contradictory to what was expected. In 2015, the respondents were older and more experienced, both of which have been shown to increase the tendency to choose a palliative care approach in recent and some previous studies including our own [47–49]. Further, understanding of palliative care in Europe and availability of education in palliative medicine in Finland have increased since 1999 [9, 50]. We suggest that our somewhat surprising result might be at least partly explained by the underrecognized needs and thresholds for palliative care in dementia compared to those of cancer as well as by the availability of less invasive diagnostic and therapeutic strategies for gastrointestinal bleeding in 2015 than 16 years earlier [51–53]. In addition, the demands for better care for elderly people by society might have had an influence on the choice of a more active approach.

We also found it surprising that GPs chose a palliative care approach less frequently than other physicians in both years. Furthermore, they chose active care more often in 2015 than in 1999 (Table 2). GPs’ unwillingness to choose a palliative care approach was also seen in our earlier study concerning decision-making for a terminally ill cancer patient [49]. It is worth mentioning that most GPs in Finland work in outpatient units, and they are not as used to emergent situations as doctors working in hospitals. It may be assumed that physicians working in hospitals are more familiar with senior patients with acute gastrointestinal bleeding and may be more aware of the prognosis in this kind of situation. However, as a majority of physicians face patients in emergency situations at least occasionally, doctors should be familiar with the common features of very advanced diseases.

Oncologists have been shown to withhold or withdraw futile treatments in EOL care and to choose palliative care more often than other physician groups [3, 41, 49]. In our current study, 77% of the oncologists chose palliative care in 1999, but this proportion declined to 56% in 2015 (Table 2). Oncologists’ general knowledge of palliative care might be greater than that in other physician groups, but despite of the increasing number of patients with coexisting cancer and dementia, dementia as a terminal illness is probably not very familiar to all of them. In addition, current targeted cancer treatment options with fewer side-effects combined with the expansion of geriatric oncology have made it possible to offer safer, more effective and more personalized therapy to older and vulnerable patients [12, 54–56]. These may at least partly explain the increased willingness of oncologists to offer a possible life-prolonging therapy in our dementia patient scenario in 2015 compared to 1999.

Older age of the physician increased the tendency to choose palliative care in our study. In other studies, the age of the physician seems to have an inconsistent influence on decision-making in EOL care [7]. Higher age usually correlates with experience, which can be expected to add confidence in decision-making. On the other hand, younger physicians may have had better or at least more education in palliative care than their older colleagues [6, 57, 58]. Nevertheless, EOL decision-making in dementia patients seems to be easier for more experienced doctors [59]. Alemayehu and co-workers studied physicians’ treatment choices using an almost identical case vignette to ours and found that physicians’ older age was associated with decreased likelihood to choose active treatment [60, 61].

The influence of the family’s benefit in decision-making decreased between 1999 and 2015, whereas the influence of the patient’s benefit and ethical values as well as the patient’s or physician’s legal protection increased. These findings are in line with those of our previous study [6]. The current tendency to value the patient’s benefit more than in 1999 may reflect the general rise of individualism and tendency to emphasize patient rights in Western countries [62, 63].

Legal issues are often perceived as a source of pressure in decision-making, and strong legal concerns may lead to more aggressive diagnostic testing and treatment choices [58, 64, 65]. Finnish law (the Act on the Status and Rights of Patients, 1992) states that the patient has to be cared for in a mutual understanding with him/her and, in the case of his/her incompetency, a representative of the patient has to be heard to assess, what kind of care and treatment would be in accordance with the patient’s will [66]. The inability to discuss the treatment options with the patient or his family in our case scenario may have increased the importance of legal aspects on respondents’ decision-making. Unfortunately, a lack of important information is common with older patients in acute care situations, which makes decision-making difficult for physicians and highlights the importance of understanding the benefits and harms of different treatment options in frail patients with advanced diseases to make reasonable choices complying with the assumed best benefit of an individual patient [4]. What comes to our patient scenario however, the importance of shared decision-making might have increased the respondents’ willingness to choose active approach in order to sustain the patient until his family can be reached and the goal of care will be discussed.

No changes in decision-making were seen regarding patient age, cost of care and patient social status. Even though high age is a well-known risk factor for poor survival after an acute illness, age alone is not seen as an acceptable reason for withholding or withdrawing life-sustaining treatment in acute care setting [67, 68]. A big challenge with the aging population involves learning to identify elderly patients who still benefit from intensive care procedures. Older people should also be encouraged to express their wishes in written format before they become seriously ill [4, 32, 69, 70]. One goal of this study was to rise discussion and pay attention to EOL care among Finnish physicians and other caregivers and encourage them to make ACP with their patients. Older people should also be encouraged to express their wishes in written format before they become seriously ill [4, 32, 69, 70]. If the patient in our scenario would have had an advance directive, it would probably have affected markedly physicians’ decision-making as in our previous studies concerning cancer patients [6, 49].

## Conclusions

Finnish physicians, especially GPs and oncologists, chose palliative care approach less often in 2015 than in 1999 for a patient with life-threatening gastrointestinal bleeding and advanced dementia. GPs were most likely to choose active/intensive care in both years. In addition to specialty, many background factors influenced the complex decision-making of a physician. Considering the aging population, all physicians treating older patients should be able to recognize advanced dementia as a life-limiting condition and to avoid burdensome interventions and futile therapies. Therefore, post- and undergraduate education concerning dementia and focusing on palliative care are needed to make well-timed decisions to shift the goal of therapy from cure to care.

## Data Availability

The datasets used and analysed during the study are available from the corresponding author on reasonable request.

## Abbreviations

ACP: Advance care planning
CFS: Clinical frailty scale
COVID-19: coronavirus disease 2019
EOL: End-of-life
GP: General practitioner

## References

[1] Prince M. World Alzheimer Report 2015. https://www.alz.co.uk/research/WorldAlzheimerReport2015.pdf. Accessed 16 Feb 2020.

[2] Mitchell SL, Teno JM, Kiely DK, Shaffer ML, Jones RND, Prigerson HG (2009). The clinical course of advanced dementia. N Engl J Med.

[3] Hinkka H, Kosunen E, Lammi UK, Metsänoja R, Puustelli A, Kellokumpu-Lehtinen P (2002). Decision making in terminal care: a survey of Finnish doctor’s treatment decisions in end-of-life scenarios involving a terminal cancer and a terminal dementia patient. Palliat Med.

[4] Guidet B, Flaatten H, Boumendil A, Morandi A, Andersen F, Artigas A (2018). Withholding or withdrawing of life-sustaining therapy in older adults (≥ 80 years) admitted to the intensive care unit. Intensive Care Med.

[5] Piili R. End-of-life decision-making in cancer patients: attitudes, ethics and background factors among Finnish physicians and medical students. Tampere University; 2019. http://urn.fi/URN:ISBN:978-952-03-1268-8. Accessed 24 May 2020.

[6] Piili RP, Lehto JT, Metsänoja R, Hinkka H, Kellokumpu-Lehtinen PLI (2019). Has there been a change in the end-of-life decision-making over the past 16 years?. BMJ Support Palliat Care.

[7] Frost DW, Cook DJ, Heyland DK, Fowler RA (2011). Patient and healthcare professional factors influencing end-of-life decision-making during critical illness: a systematic review. Crit Care Med.

[8] Singer AE, Goebel JR, Kim YS, Dy SM, Ahluwalia SC, Clifford M (2016). Populations and interventions for palliative and end-of-life care: a systematic review. J Palliat Med.

[9] The Finnish Medical Association. Special Education: Lääkäriliitto - Palliatiivinen lääketiede. https://www.laakariliitto.fi/palvelut/koulutukset/erityispatevyydet/palliatiivinen/. Accessed 10 Jan 2020.

[10] Shepherd H, Livingston G, Chan J, Sommerlad A. Hospitalisation rates and predictors in people with dementia: a systematic review and meta-analysis. BMC Med. 2019;17(1):1–13. 10.1186/s12916-019-1369-7 PMC6628507 31303173

[11] Barclay S, Froggatt S, Crang C, Mathie E, Iliffe S, Manthorpe J (2014). Living in uncertain times: trajectories to death in residential care homes. Br Gen Pract.

[12] Kenis C, Decoster L, Flamaing J, Debruyne PR, de Groof I, Focan C (2018). Adherence to geriatric assessment-based recommendations in older patients with cancer: a multicenter prospective cohort study in Belgium. Ann Oncol.

[13] Gupta Mohile S, Velarde C, Hurria A, Magnuson A, Lowenstein L, Pandya C (2015). Geriatric assessment-guided care processes for older adults: a delphi consensus of geriatric oncology experts. J Natl Compr Canc Netw.

[14] Wildiers H, Heeren P, Puts M, Topinkova E, Janssen-Heijnen ML, Extermann M (2014). International society of geriatric oncology consensus on geriatric assessment in older patients with cancer purpose to update the International Society of Geriatric Oncology (SIOG) 2005 recommendations on geriatric assessment (GA) in older patients with c. J Clin Oncol.

[15] Mohile SG, Dale W, Somerfield MR, Schonberg MA, Boyd CM, Burhenn PS (2018). Practical assessment and management of vulnerabilities in older patients receiving chemotherapy: Asco guideline for geriatric oncology. J Clin Oncol.

[16] Lee EE, Chang B, Huege S, Hirst JE (2018). Complex clinical intersection: palliative care in patients with dementia. Am J Geriatr Psychiatry.

[17] Chen JH, Chan DC, Kiely DK, Morris JN, Mitchell SL (2007). Terminal trajectories of functional decline in the long-term care setting. J Gerontol A Biol Sci Med Sci.

[18] Pickens S, Naik AD, Catic A, Kunik ME (2017). Dementia and hospital readmission rates: a systematic review. Dement Geriatr Cogn Disord Extra.

[19] Gozalo P, Teno JM, Mitchell SL, Skinner J, Bynum J, Tyler D (2011). End-of-life transitions among nursing home residents with cognitive issues. N Engl J Med.

[20] Livingston G, Huntley J, Sommerlad A, Ames D, Ballard C, Banerjee S (2020). Dementia prevention, intervention, and care: 2020 report of the Lancet Commission. Lancet.

[21] Dixon J, Karagiannidou M, Knapp M (2018). The effectiveness of advance care planning in improving end-of-life outcomes for people with dementia and their carers: a systematic review and critical discussion. J Pain Symptom Manage.

[22] Saarto T, Finne-Soveri H, and expert working group. Suositus palliatiivisen hoidon palveluiden tuottamisesta ja laadun parantamisesta Suomessa. Palliatiivisen hoidon asiantuntijaryhmän loppuraportti. Reports and Memorandums of the Ministry of Social Affairs and Health. 2019;68. http://julkaisut.valtioneuvosto.fi/handle/10024/161946. Accessed 18 Feb 2020.

[23] Sampson EL, Candy B, Davis S, Gola AB, Harrington J, King M (2018). Living and dying with advanced dementia: a prospective cohort study of symptoms, service use and care at the end of life. Palliat Med.

[24] Brown MA, Sampson EL, Jones L, Barron AM (2013). Prognostic indicators of 6-month mortality in elderly people with advanced dementia: a systematic review. Palliat Med.

[25] Erel M, Marcus E-L, Dekeyser-Ganz F (2017). Barriers to palliative care for advanced dementia: a scoping review. Ann Palliat Med.

[26] Murphy E, Froggatt K, Connolly S, O’Shea E, Sampson EL, Casey D (2016). Palliative care interventions in advanced dementia. Cochrane Database Syst Rev.

[27] Mitchell SL (2015). Advanced dementia. N Engl J Med.

[28] Thomas KS, Belanger E, Zhang W, Carder P (2020). State variability in assisted living residents’ end-of-life care trajectories. J Am Med Dir Assoc.

[29] Teno JM, Gozalo P, Trivedi AN, Bunker J, Lima J, Ogarek J (2018). Site of death, place of care, and health care transitions among US medicare beneficiaries, 2000–2015. JAMA.

[30] Konttila T, Finne-Soveri UH, Leskinen R, Niemelä K, Antikainen R (2020). Progress in advance care planning among nursing home residents dying with advanced dementia—does it make any difference in end-of-life care?. Arch Gerontol Geriatr.

[31] van Riet PJ, Mariani E, Chattat R, Koopmans R, Kerhervé H, Leppert W (2015). Identification of the palliative phase in people with dementia: a variety of opinions between healthcare professionals. BMC Palliat Care.

[32] Dening K, Sampson EL, de Vries K (2019). Advance care planning in dementia: recommendations for healthcare professionals. Palliat Care.

[33] Vandervoort A , Houttekier D, vander Stichele R, van der Steen JT, van den Block L (2014). Quality of dying in nursing home residents dying with dementia: does advanced care planning matter? A nationwide postmortem study. PLoS ONE.

[34] Sellars M, Chung O, Nolte L, Tong A, Pond D, Fetherstonhaugh D (2019). Perspectives of people with dementia and carers on advance care planning and end-of-life care: a systematic review and thematic synthesis of qualitative studies. Palliat Med.

[35] Mitchell SL, Kiely DK, Hamel MB (2004). Dying with advanced dementia in the nursing home. Arch Intern Med.

[36] Vandervoort A, van den Block L, van der Steen JT, van der Stichele R, Bilsen J, Deliens L (2012). Advance directives and physicians’ orders in nursing home residents with dementia in Flanders, Belgium: prevalence and associated outcomes. Int Psychogeriatr.

[37] Garand L, Amanda Dew M, Lingler JH, DeKosky ST (2011). Incidence and predictors of advance care planning among persons with cognitive impairment. Am J Geriatr Psychiatry.

[38] Fallon A, Dukelow T, Kennelly SP, O’neill D (2020). COVID-19 in nursing homes. QJM.

[39] British Geriatrics Society. COVID-19: Managing the COVID-19 pandemic in care homes | British Geriatrics Society. 2020 https://www.bgs.org.uk/resources/covid-19-managing-the-covid-19-pandemic-in-care-homes. Accessed 2 Aug 2020.

[40] Perrotta F, Corbi G, Mazzeo G, Boccia M, Aronne L, D’Agnano V (2020). COVID-19 and the elderly: insights into pathogenesis and clinical decision-making. Aging Clin Exp Res.

[41] Hinkka H, Kosunen E, Metsänoja R, Lammi UK, Kellokumpu-Lehtinen P (2002). Factors affecting physicians’ decisions to forgo life-sustaining treatments in terminal care. J Med Ethics.

[42] Piili RP, Metsänoja R, Hinkka H, Kellokumpu-Lehtinen P-LI, Lehto JT (2018). Changes in attitudes towards hastened death among Finnish physicians over the past sixteen years. BMC Med Ethics.

[43] The Finnish Medical Association. Physicians in Finland statistics on physicians and the health care system 2016. The Finnish Medical Association. https://www.laakariliitto.fi/site/assets/files/5223/ll16_tilasto2016_net1_170114.pdf. Accessed 15 Apr 2021.

[44] Rockwood K, Song X, MacKnight C, Bergman H, Hogan DB, McDowell I (2005). A global clinical measure of fitness and frailty in elderly people. CMAJ.

[45] Dent E, Kowal P, Hoogendijk EO (2016). Frailty measurement in research and clinical practice: a review. Eur J Intern Med.

[46] Temel JS, Greer JA, El-Jawahri A, Pirl WF, Park ER, Jackson VA (2017). Effects of early integrated palliative care in patients with lung and gi cancer: a randomized clinical trial. J Clin Oncol.

[47] Larochelle MR, Rodriguez KL, Arnold RM, Barnato AE (2009). Hospital staff attributions of the causes of physician variation in end-of-life treatment intensity. Palliat Med.

[48] Löfmark R, Nilstun T, Cartwright C, Fischer S, van der Heide A, Mortier F (2008). Physicians’ experiences with end-of-life decision-making: survey in 6 European countries and Australia. BMC Med.

[49] Piili RP, Lehto JT, Luukkaala T, Hinkka H, Kellokumpu-Lehtinen P-LI (2018). Does special education in palliative medicine make a difference in end-of-life decision-making?. BMC Palliat Care.

[50] Connor SR. Global Atlas of Palliative Care. Worldwide Hospice Palliative Care Alliance, 2nd ed. World Health Organization; 2020. http://www.thewhpca.org/resources/global-atlas-on-end-of-life-care. Accessed 9 Aug 2020.

[51] Lhewa DY, Strate LL (2012). Pros and cons of colonoscopy in management of acute lower gastrointestinal bleeding. World J Gastroenterol.

[52] Feinman M, Haut ER (2014). Upper gastrointestinal bleeding. Surg Clin North Am.

[53] Shah AR, Jala V, Arshad H, Bilal M (2018). Evaluation and management of lower gastrointestinal bleeding. Dis Mon.

[54] Kelly CM, Power DG, Lichtman SM (2014). Targeted therapy in older patients with solid tumors. J Clin Oncol.

[55] Swaminathan D, Swaminathan V (2015). Geriatric oncology: problems with under-treatment within this population. Cancer Biol Med.

[56] Wildiers H, Heeren P, Puts M, Topinkova E, Janssen-Heijnen MLG, Extermann M (2014). International society of geriatric oncology consensus on geriatric assessment in older patients with cancer. J Clin Oncol.

[57] Bopp M, Penders YWH, Hurst SA, Bosshard G, Puhan MA (2018). Physician-related determinants of medical end-of-life decisions – a mortality follow-back study in Switzerland. PLoS ONE.

[58] Forte DN, Vincent JL, Velasco IT, Park M (2012). Association between education in EOL care and variability in EOL practice: a survey of ICU physicians. Intensive Care Med.

[59] McDermott C, Coppin R, Little P, Leydon G (2012). Hospital admissions from nursing homes: a qualitative study of GP decision making. Br J Gen Pract.

[60] Alemayehu E, Molloy DW, Guyatt GH, Singer J, Penington G, Basile J (1991). Variability in physicians’ decisions on caring for chronically ill elderly patients: an international study. CMAJ.

[61] Molloy DW, Guyatt GH, Alemayehu E, Mcnlroy W, Willan A, Eisemann M (1991). Factors affecting physicians’ decisions on caring for an incompetent elderly patient: an international study. CMAJ.

[62] Twenge JM, Campbell WK, Gentile B (2012). Increases in individualistic words and phrases in American books, 1960–2008. PLoS One.

[63] Greenfield PM (2013). The changing psychology of culture from 1800 through 2000. Psychol Sci.

[64] Carrier ER, Reschovsky JD, Katz DA, Mello MM (2013). High physician concern about malpractice risk predicts more aggressive diagnostic testing in office-based practice. Health Aff (Millwood).

[65] Reschovsky JD, Saiontz-Martinez CB (2018). Malpractice claim fears and the costs of treating medicare patients: a new approach to estimating the costs of defensive medicine health services research. Health Serv Res.

[66] Laki potilaan asemasta ja oikeuksista 785/1992 - Ajantasainen lainsäädäntö - FINLEX ®. https://www.finlex.fi/fi/laki/ajantasa/1992/19920785. Accessed 17 Jan 2021.

[67] Guidet B, Vallet H, Boddaert J, de Lange DW, Morandi A, Leblanc G (2018). Caring for the critically ill patients over 80: a narrative review. Ann Intensive Care.

[68] de Lange DW, Brinkman S, Flaatten H, Boumendil A, Morandi A, Andersen FH (2019). Cumulative prognostic score predicting mortality in patients older than 80 years admitted to the ICU. J Am Ger Soc.

[69] Piers R, Albers G, Gilissen J, de Lepeleire J, Steyaert J, van Mechelen W (2018). Advance care planning in dementia: recommendations for healthcare professionals. BMC Palliat Care.

[70] van der Steen JT, Radbruch L, Hertogh CM, de Boer ME, Hughes JC, Larkin P (2014). White paper defining optimal palliative care in older people with dementia: a Delphi study and recommendations from the European Association for Palliative Care on behalf of the European Association for Palliative Care (EAPC). Palliat Med.

